# Genomic Diversity of a Globally Used, Live Attenuated Mycoplasma Vaccine

**DOI:** 10.1128/spectrum.02845-22

**Published:** 2022-11-01

**Authors:** Sara M. Klose, Olusola M. Olaogun, Jillian F. Disint, Pollob Shil, Miklós Gyuranecz, Zsuzsa Kreizinger, Dorottya Földi, Salvatore Catania, Marco Bottinelli, Arianna Dall'Ora, Anneke Feberwee, Marleen van der Most, Daniel M. Andrews, Gregory J. Underwood, Chris J. Morrow, Amir H. Noormohammadi, Marc S. Marenda

**Affiliations:** a Asia Pacific Centre for Animal Health, Melbourne Veterinary School, The University of Melbourne, Victoria, Australia; b Bioproperties Pty Ltd., Victoria, Australia; c Veterinary Medical Research Institute, Eötvös Loránd Research Network, Budapest, Hungary; d MolliScience Kft., Biatorbágy, Hungary; e Mycoplasma Unit, SCT-1 Verona, Istituto Zooprofilattico Sperimentale delle Venezie, Buttapietra, Italy; f Royal GD, Deventer, the Netherlands; g Wageningen Bioveterinary Research, Wageningen University and Research, Lelystad, The Netherlands; Houston Methodist Hospital

**Keywords:** mycoplasma, whole-genome sequencing, live vaccine

## Abstract

The Mycoplasma synoviae live attenuated vaccine strain MS-H (Vaxsafe MS; Bioproperties Pty., Ltd., Australia) is commonly used around the world to prevent chronic infections caused by M. synoviae in birds and to minimize economic losses in the poultry industry. MS-H is a temperature-sensitive strain that is generated via the chemical mutagenesis of a virulent M. synoviae isolate, 86079/7NS. 32 single nucleotide polymorphisms have been found in the genome of MS-H compared to that of 86079/7NS, including 25 in predicted coding sequences (CDSs). There is limited information on the stability of these mutations in MS-H *in vitro* during the propagation of the vaccine manufacturing process or *in vivo* after the vaccination of chickens. Here, we performed a comparative analysis of MS-H genomes after *in vitro* and *in vivo* passages under different circumstances. Studying the dynamics of the MS-H population can provide insights into the factors that potentially affect the health of vaccinated birds. The genomes of 11 *in vitro* laboratory passages and 138 MS-H bird reisolates contained a total of 254 sequence variations. Of these, 39 variations associated with CDSs were detected in more than one genome (range = 2 to 62, median = 2.5), suggesting that these sequences are particularly prone to mutations. From the 25 CDSs containing previously characterized variations between MS-H and 86079/7NS, 7 were identified in the MS-H reisolates and progenies examined here. In conclusion, the MS-H genome contains individual regions that are prone to mutations that enable the restoration of the genotype or the phenotype of wild-type 86079/7NS in those regions. However, accumulated mutations in these regions are rare.

**IMPORTANCE** Preventative measures, such as vaccination, are commonly used for the control of mycoplasmal infections in poultry. A live attenuated vaccine strain (Vaxsafe MS; MS-H; Bioproperties Pty. Ltd., Australia) is used for the prevention of disease caused by M. synoviae in many countries. However, information on the stability of previously characterized mutations in the MS-H genome is limited. In this study, we performed a comparative analysis of the whole-genome sequences of MS-H seeds used for vaccine manufacturing, commercial batches of the vaccine, cultures minimally passaged under small-scale laboratory and large-scale manufacturing conditions, MS-H reisolated from specific-pathogen-free (SPF) chickens that were vaccinated under controlled conditions, and MS-H reisolated from vaccinated commercial poultry flocks around the world. This study provides a comprehensive assessment of genome stability in MS-H after *in vitro* and *in vivo* passages under different circumstances and suggests that most of the mutations in the attenuated MS-H vaccine strain are stable.

## INTRODUCTION

Mycoplasmas have the smallest cell size and genome of all self-replicating prokaryotes (down to 300 nm in diameter and 580 kb) (1). Mycoplasmas occur worldwide as pathogenic and nonpathogenic species of many animals, including poultry (2). Avian mycoplasmosis was first identified in 1926 (3), and the first reports of infectious synovitis and airsacculitis caused by Mycoplasma synoviae were described in 1956 and 1973, respectively (4, 5). Synovitis in turkeys and respiratory disease in chickens caused by M. synoviae are responsible for economic losses in the poultry industry, and these are compounded by the chronic nature of these infections (6, 7), resulting in a decrease in egg production and quality, poor hatchability, and poor feed conversion, as well as an increase in mortality rates (3, 8, 9). Moreover, the treatment of M. synoviae infections often requires long-term antibiotic use, increasing the likelihood of the emergence of antimicrobial resistance (10–12). The live attenuated vaccine strain MS-H (Vaxsafe MS; Bioproperties Pty., Ltd., Australia) is used for the prevention of M. synoviae infections in many countries. MS-H is a temperature-sensitive (*ts^+^*) clone derived from a virulent field strain of M. synoviae, 86079/7NS, referred to here as 7NS (13–15). The chemical mutagen N-methyl-N0-nitro-N-nitrosoguanidine (NTG) was used to create MS-H. NTG mutants are thought to be prone to a reversion to virulence (16, 17). The molecular mechanisms underpinning the *ts^+^* phenotypes have been investigated in various bacteria and viruses (18–22). To explore the genetic bases of virulence attenuation and temperature sensitivity in MS-H, the complete genome sequence of this strain (GenBank accession number CP021129) was compared to that of its parent 7NS (GenBank accession number CP029258.1), which revealed 32 single nucleotide polymorphisms (SNPs) (23). A genome analysis of M. synoviae isolates that were recovered from vaccinated flocks identified that only a few of the mutations in the MS-H strain have reverted to the parent strain sequence (24). Studying the genomic stability of the live MS-H strain and the dynamics of the population after vaccination will improve our understanding on whether there are any clues regarding factors that could potentially influence the health of vaccinated or in-contact, naive birds. Here, we compared the whole-genome sequences of MS-H clones, collected mostly from vaccinated poultry farms in 10 countries (referred to here as “field reisolates”) from specific-pathogen-free (SPF) chickens that were vaccinated and kept in isolators under controlled conditions (referred to here as “controlled reisolates”), SPF chickens inoculated with cultures from previously vaccinated birds to produce consecutive *in vivo* passages under controlled conditions (referred to here as “passaged reisolates”), and MS-H clones obtained after six *in vitro* passages in a 10 mL volume (referred to here as “small-scale progenies”) or in commercial manufacturing fermenters (referred to here as “large-scale progenies”). We described the variations detected among these genomes. This study provides a comprehensive assessment of the genomic variations in MS-H after *in vivo* and *in vitro* passages and suggests that most of the mutations developed in the 7NS virulent isolate to obtain the MS-H vaccine strain are stable. We also characterized several sequence changes in the MS-H genome that enable the restoration of a parental 7NS genotype in some genes, and we evaluated their potential impact on the functions of the corresponding proteins.

## RESULTS

### The MS-H genome is stable after minimal *in vitro* and *in vivo* passages.

We first investigated the variability of MS-H that was directly provided by the manufacturer. Genome alignments of a Vaxsafe MS master seed (*n* = 1), a working seed (*n* = 1), and commercial batches (*n* = 4) to that of the published MS-H sequence (GenBank accession number CP021129), excluding the repetitive and variable regions, did not reveal any sequence variation. In individual colonies randomly picked from plated cultures of samples to represent the different steps of the production of a vaccine batch, two out of three colonies from the first stage as well as three out of three colonies from the final stage were identical to the MS-H genome sequence. Only one colony from the first stage of vaccine production contained a SNP leading to an amino acid change from alanine to valine at position 156 of a putative P80-related lipoprotein (MSH_RS01430) (Table 1).

**TABLE 1:** Single nucleotide polymorphisms detected in isolates obtained after *in vitro* and *in vivo* passages^*a*^

Culture condition	Isolate ID	Reference nucleotide (MS-H)	Variant nucleotide	Strand	Effect on coding	Variant type	Locus-tag	Product
*In vitro* large-scale	BPL-1-2	G	A	−	Ala156Val	Missense	MSH_RS01430	P80 family lipoprotein
*In vitro* small-scale	Vit-6-2	G	A	−	Ala156Val	Missense	MSH_RS01430	P80 family lipoprotein
	Vit-6-4	G	A	−	Ala156Val	Missense	MSH_RS01430	P80 family lipoprotein
	Vit-6-5	C	A	−	Glu329*	Stop gained	MSH_RS02365	DUF31 family protein
	Vit-6-1	A	G	−	Ala175Ala	Synonymous	MSH_RS01870	GNAT family N-acetyltransferase
*In vivo*	Viv-4-1	G	A	−	Ser486Ser	Synonymous	MSH_RS02650	TrkH family potassium uptake protein
	Viv-4-2	G	A	−	Ser486Ser	Synonymous	MSH_RS02650	TrkH family potassium uptake protein
	Viv-5-2	G	A	−	Ser486Ser	Synonymous	MSH_RS02650	TrkH family potassium uptake protein
	Viv-5-5	G	A	−	Ser486Ser	Synonymous	MSH_RS02650	TrkH family potassium uptake protein
	Viv-5-6	G	A	−	Ser486Ser	Synonymous	MSH_RS02650	TrkH family potassium uptake protein

a BPL-1-2, colony number 2 picked from plated cultures of samples representing the first stage of vaccine production; Vit-6-1, 2, 4, 5, random colonies picked from plated cultures of the sixth *in vitro* passage under laboratory conditions; Viv-4-1, 2, colonies picked from plated tracheal washing/nasal turbinate mixtures of the chickens in passage 4; Viv-5-2, 5, 6, colonies picked from plated tracheal washing/nasal turbinate mixtures of the chickens in passage 5; *, stop codon.

To confirm these observations, we then assessed the genomic stability of an aliquot of the stock culture of MS-H that was used to establish the publicly available sequence CP021129 and was kept in our laboratory (UoM_MS-H). Out of five colonies obtained after six *in vitro* passages of UoM_MS-H, the genome of 1 colony was identical to that of the published reference MS-H genome. The genomes of two colonies contained a SNP in the P80-related lipoprotein, identical to the one detected in the above-mentioned clone variant from the vaccine production. Two other colonies obtained from the sixth *in vitro* passage contained other variations, as follows. In one of the colonies, a single nucleotide polymorphism resulted in a premature stop codon and a truncation of the DUF31 family protein. In another colony, a synonymous substitution was found in a putative GCN5-related N-acetyltransferase (GNAT) family N-acetyltransferase (Table 1).

Finally, we examined the variability of MS-H over consecutive rounds of short-term propagation in SPF birds that were kept in controlled conditions. Chickens were vaccinated with a commercial MS-H preparation (batch number 250294-2), and the live vaccine was passaged in chickens five times by inoculating new birds with fresh cultures obtained from the previous group of birds shortly after being vaccinated. A total number of 15 colonies (3 per passage) cultivated from the tracheal washing/nasal turbinate mixtures of the chickens in each passage were sequenced. The genome sequences of these colonies were aligned to the one from the initial MS-H batch 250294-2, excluding the repetitive and variable regions. Only one variation was detected in two colonies obtained from passage four and in three colonies obtained from passage five, all of which affected the nucleotide position at 615106 and resulted in a synonymous codon change in the TrkH family potassium uptake protein (MSH_RS02650), which did not affect the protein sequence (Table 1).

### Variations in the MS-H genomes collected from experimentally vaccinated chickens suggest that MS-H undergoes limited mutations in the natural host over time.

Since minimal, short-term, *in vitro* and *in vivo* passages did not appear to create widespread genome variations of MS-H, we explored the variability of the vaccine over a longer period of time in birds so as to simulate on-farm conditions. Out of five chickens vaccinated with the previously sequenced MS-H working seed (BPL-157), no Mycoplasma growth was observed in the swabs collected from the middle or lower trachea. In contrast, MS-H was recovered from all of the swabs collected from the upper trachea of all five of the chickens at 60 days postinoculation. The sequenced genomes, excluding the repetitive and variable regions, of 25 colonies (5 per bird) were aligned to that of the MS-H working seed strain that was used to vaccinate the chickens in this experiment. The sequence analysis results are summarized in Table 2. The most frequent variation was detected in three different chickens (chicken ID numbers 728, 729, 730) as a variation leading to an amino acid change from alanine to valine at position 156 of a putative P80 related lipoprotein (MSH_RS01430). Several other coding regions contained variations in this experiment: two putative immunoglobulin-blocking virulence proteins, a DNA-directed RNA polymerase, a putative major facilitator superfamily (MFS) transporter, *N*-acetylglucosamine-6-phosphate deacetylase, GTPase Era, and an UvrD-helicase domain-containing protein. Two other sequenced genomes collected from chicken 730 and 738 contained further variations that resulted in amino acid changes in the GTPase ObgE protein and DNA-directed RNA polymerase, respectively, changing both of these protein sequences to those of 7NS. Three colonies obtained from chicken 729 contained a synonymous codon change in the RNA polymerase sigma factor and thus had no effect on the protein sequence.

**TABLE 2:** Variations detected in MS-H reisolates collected from vaccinated chickens under laboratory conditions^*a*^

Nucleotide position	Variation type^*b*^	Reference nucleotide (MS-H)	Variant nucleotide	Strand	Effect on coding	Variant type	Locus-tag	Product	Isolate ID		
316188	snp	G	A	−	Ala156Val	Missense	MSH_RS01430	P80 family lipoprotein	728-14	729-21	730-13
519251	snp	A	G	+	Leu42Leu	Synonymous	MSH_RS03695	RNA polymerase sigma factor	729-11	729-12	729-25
551032	snp	C	G	−	Glu190Gln	Missense	MSH_RS02405	Immunoglobulin-blocking virulence protein	734-13	738-11	738-22
573223	del	ATGG	A	−	Thr638del	In-frame Deletion	MSH_RS02470	DNA-directed RNA polymerase subunit beta	734-13	738-11	738-22
73465	snp	G	A	−	Ala561Val	Missense	MSH_RS00395	Immunoglobulin-blocking virulence protein	734-12	734-22	
562736	snp	C	A	−	Trp473Leu	Missense	MSH_RS02450	Major facilitator superfamily transporter	734-13	738-11	
193771	snp	A^*b*^	G^*b*^	+	Arg123Gly	Missense	MSH_RS00965	GTPase ObgE	730-13		
235142	del	TA	T	+	Ile108fs	Frameshift	MSH_RS01120	N-acetylglucosamine-6-phosphate deacetylase	734-21		
444843	snp	G	C	−	Ser95Trp	Missense	MSH_RS01960	GTPase Era	738-23		
567729	snp	A^*b*^	C^*b*^	−	Ile1270Arg	Missense	MSH_RS02465	DNA-directed RNA polymerase subunit beta	738-12		
644452	snp	G	C	−	His715Asp	Missense	MSH_RS02780	UvrD-helicase domain-containing protein	738-12		

a del, deletion; snp, single nucleotide polymorphism; fs, frameshift.

b The indicated variations have been previously described between MS-H and 7NS.

### The MS-H reisolates from poultry farms possess a small number of common variations in coding regions.

**FIG 1:**
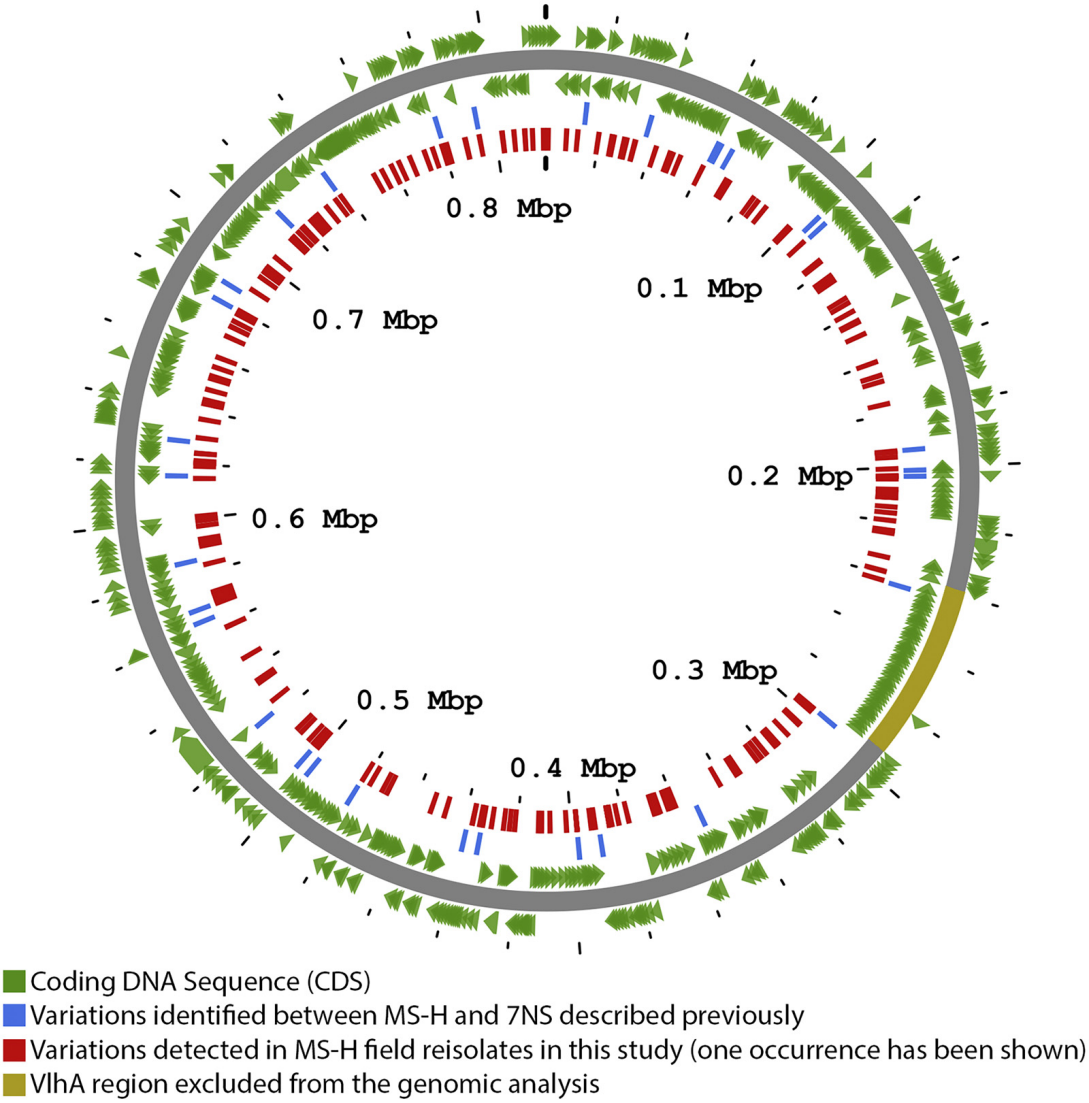
Map of the sequence variations detected in MS-H field reisolates. Coding DNA sequences of the MS-H genome are shown in green. The 253 variations detected in the 98 sequenced genomes of MS-H field reisolates, following the alignment with the MS-H genome, are shown in red. Variations shown in blue have been previously described between MS-H and 7NS. The VlhA region, which was excluded from the genomic analysis, is highlighted in yellow. The figure was made using Proksee (https://proksee.ca).

Since the vaccine industrial production processes and vaccinations of birds kept in controlled experimental conditions did not appear to lead to widespread genomic changes, we sought to evaluate the stability of MS-H in field conditions. We sequenced a wide collection of M. synoviae strains that were isolated from commercial poultry farms in which birds had been previously vaccinated with MS-H. The comparison of 98 field reisolates with the reference MS-H genome revealed 253 variations, from which 182 were detected in 132 predicted coding regions (Fig. 1). A total of 39 coding regions contained variations in more than one genome, indicating variations other than spontaneous mutations (Table S3).

7 coding regions contained variations in 10 or more field reisolates identified as coding regions that were prone to mutation under different conditions (Table 3). Within 98 sequenced field reisolates, the most frequently observed coding region that was prone to mutation was locus MSH_RS01740, coding for a putative ATP-binding cassette (ABC) transporter (OppF) and containing variations in 62 reisolates. Two recurrent variations were detected in this region as a T insertion at position 397777 and an A insertion at position 397775, leading to a codon frameshift. The locus MSH_RS00965, coding for a putative GTPase (ObgE), contained variations in 54 field reisolates (Table 3). While the most frequent variation resulted in a conservative amino acid substitution at position 210 from alanine to valine, another frequent variation resulted in an amino acid substitution from arginine to glycine at amino acid position 123. The amino acid 123 was affected by a G insertion in 7 further reisolates, leading to a frameshift in the ObgE protein sequence. The third most frequent coding region prone to mutations was locus MSH_RS01365, encoding Type I glyceraldehyde-3-phosphate dehydrogenase (GAPDH) and containing variations in 13 reisolates as a single nucleotide polymorphism (SNP) at position 296526 and resulting in a conservative mutation, with an amino acid substitution from lysine to arginine at position 306 of the protein (Table 3). The region encoding a putative DNA topoisomerase IV subunit A (ParC, MSH_RS00320) contained different variations in several reisolates, including a G to A SNP at position 60458 that was observed in 5 reisolates, a C to T SNP at position 60462 that was observed in 2 reisolates, and a G to A SNP at position 60473 that was observed in 7 reisolates, resulting in Asp84Asn, Thr85Ile, and Asp89Asn substitutions in the amino acid sequences, respectively (Table 3). There were 12 variations detected in the locus MSH_RS02465, coding for the putative subunit beta of a DNA-directed RNA polymerase (Table 3). An identical variation was found in this region in 7 reisolates as a SNP at position 570580, changing the amino acid alanine to threonine at position 320 of the subunit beta of the DNA-directed RNA polymerase (Table 3). The locus MSH_RS01430, coding for a putative P80 family lipoprotein, was among the coding regions prone to mutation and contained variations in 12 field reisolates, of which 5 were identical as a SNP at position 315555 and resulted in a conservative amino acid change from alanine to valine (Table 3). Another putative sugar ABC transporter (MSH_RS00575) contained different variations that were detected in 10 field reisolates and affected a stop codon at position 219. 3 of these variations were detected as SNPs at position 107372 and led to a synonymous codon change that retained the stop codon. However, the remaining 7 occurred as A to T, A to G, or A to C at position 107373, and all of these resulted in the replacement of the stop codon by an amino acid and the extension of the protein.

**TABLE 3:** Frequent variations (more than one occurrence) detected in the mutation-prone coding regions in the MS-H field reisolates^*a*^

Locus-tag	Product	Nucleotide position	Variation type	Reference nucleotide (MS-H)	Variant nucleotide	Strand	Effect on coding	Variant type	Number of field reisolates containing variation (out of 98)
MSH_RS01740	ABC transporter, OppF	397777	ins	G^*b*^	GT^*b*^	−	Asn156fs	Frameshift	49
		397775	ins	T	TA	−	Ter157fs	Frameshift	11
		397776	ins	A	AG	−	Ter157fs	Frameshift	2
MSH_RS00965	GTPase, ObgE	194033	snp	C	T	+	Ala210Val	Missense	33
		193771	snp	A^*b*^	G^*b*^	+	Arg123Gly	Missense	12
		193771	ins	A	AG	+	Arg124fs	Frameshift	7
		194084	snp	T	A	+	Ile227Asn	Missense	2
MSH_RS01365	Glyceraldehyde-3-phosphate dehydrogenase, GAPDH	296526	snp	A^*b*^	G^*b*^	+	Lys306Arg	Missense	13
MSH_RS00320	DNA topoisomerase IV subunit A, ParC	60473	snp	G	A	+	Asp89Asn	Missense	7
		60458	snp	G	A	+	Asp84Asn	Missense	5
		60462	snp	C	T	+	Thr85Ile	Missense	2
MSH_RS02465	DNA-directed RNA polymerase subunit beta	570580	snp	C	T	−	Ala320Thr	Missense	6
		567729	snp	A	C	−	Ile1270Arg	Missense	2
MSH_RS01430	P80 family lipoprotein	315555	snp	G	A	−	Ala367Val	Missense	5
		316188	snp	G	A	−	Ala156Val	Missense	5
		316585	del	AT	A	−	Ser24fs	Frameshift	2
MSH_RS00575	Sugar ABC transporter	107372	snp	T	C	−	Ter219Ter	Stop_retained	3
		107373	snp	A	G	−	Ter219Glu	Stop_lost	3
		107373	snp	A	T	−	Ter219Lys	Stop_lost	2
		107373	snp	A	C	−	Ter219Gln	Stop_lost	2

a Mutation-prone coding regions are the coding regions manifesting variations in 10 or more field reisolates.

b The indicated variations have been described previously between MS-H and 7NS. Ins, insertion; del, deletion; snp, single nucleotide polymorphism; fs, frameshift.

### The most frequently observed mutations in MS-H lead to putative functional changes in metabolically important proteins.

The nucleotide positions showing variation in more than 10 reisolates in this study (including reisolates obtained in the *in vitro* passage, *in vivo* passage, vaccination of SPF birds under controlled conditions, and field reisolation experiments) were identified as “hotspot” regions in the MS-H genome (Table 4).

**TABLE 4:** MS-H hotspots with variation occurrence in more than 10 reisolates in this study

Nucleotide position	Variation type	Reference nucleotide (MS-H)	Variant nucleotide	Strand	Effect on coding	Variant type	Locus-tag	Product	Number of genomes containing variation (out of 143)
397777	ins	G^*a*^	GT^*a*^	−	Asn156fs	Frameshift	MSH_RS01740	ABC transporter, OppF	49
194033	snp	C	T	+	Ala210Val	Missense	MSH_RS00965	GTPase, ObgE	33
296526	snp	A^*a*^	G^*a*^	+	Lys306Arg	Missense	MSH_RS01365	Glyceraldehyde-3-phosphate dehydrogenase, GAPDH	13
193771	snp	A^*a*^	G^*a*^	+	Arg123Gly	Missense	MSH_RS00965	GTPase, ObgE	13
397775	ins	T	TA	−	Ter157fs	Frameshift	MSH_RS01740	ABC transporter, OppF	11
316188	snp	G	A	−	Ala156Val	Missense	MSH_RS01430	P80 family lipoprotein	11

a The indicated variations have been previously described between MS-H and 7NS. Ins, insertion; del, deletion; snp, single nucleotide polymorphism; fs, frameshift.

**FIG 2:**
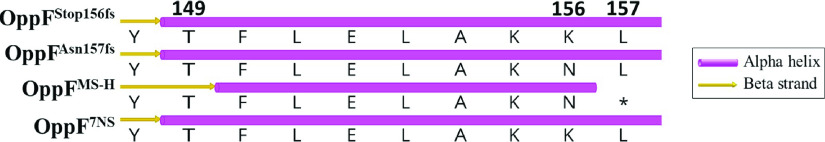
Comparison of amino acid sequences of variants detected in the OppF protein to those of MS-H and 7NS. The variations at position 157 of the OppF protein that were detected in 11 MS-H reisolates (OppF^Stop157fs^) resulted in the loss of the stop codon after the Arg-156-Lys substitution and the resumption of the parental reading frame, whereas the variations at position 156 of this protein that were detected in most of the MS-H reisolates (OppF^Asn156fs^) resulted in loss of the stop codon and the reversion of the OppF amino acid sequence to that of 7NS. *, stop codon; fs: frameshift.

Two frequent variations in the OppF protein were observed only among the field reisolates and occurred as a T or an A insertion at position 397777 or 397775, respectively. Both of these variations resulted in loss of the stop codon and, therefore, the full-length expression of the OppF protein. The amino acid sequence of the OppF^Stop156fs^ variant is identical to that of the MS-H parent strain, 7NS (Fig. 2). Based on a Uniprot analysis, the closest homologous protein to OppF of M. synoviae was identified in M. arginini, which displayed a sequence identity of 77% and encoded a putative peptide ATP-binding cassette (ABC) transporter.

**FIG 3:**
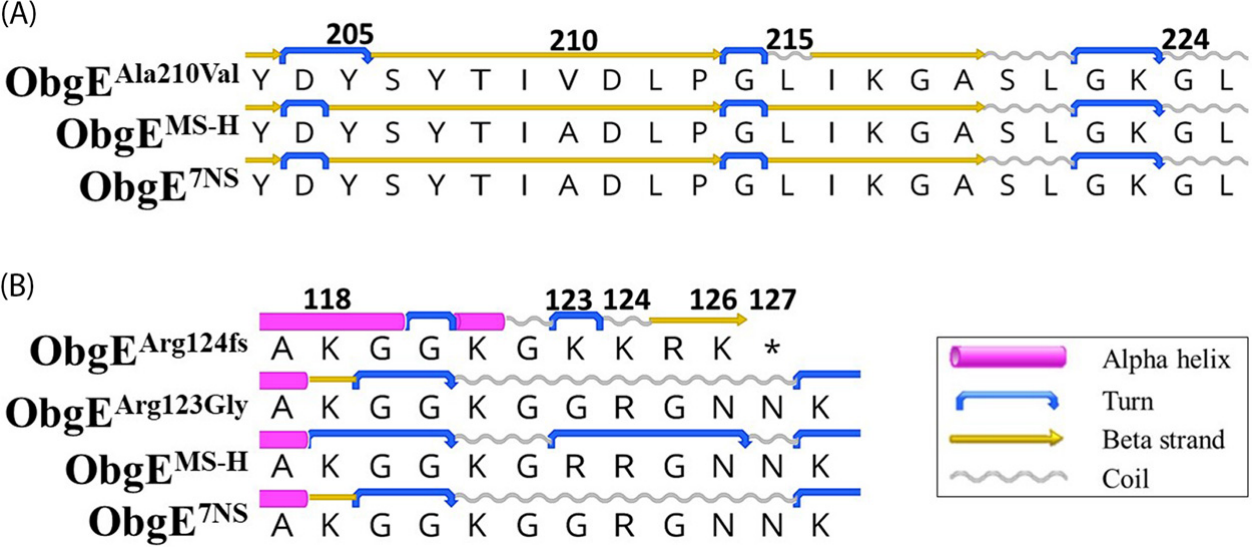
Comparison of the amino acid sequences of the most frequent variants of GTPase ObgE. (A) The effect of an Ala210Val substitution on the secondary structure of the GTPase ObgE protein of MS-H reisolates (ObgE^Ala210Val^) compared to that of MS-H. (B) The variations at position 123 of the ObgE protein that were detected in 12 MS-H reisolates (ObgE^Arg123Gly^) resulted in a reversion to the amino acid sequence of 7NS, whereas the variations at position 124 of this protein that were detected in 7 MS-H reisolates (ObgE^Arg124fs^) resulted in a frameshift and the introduction of a stop codon. *, stop codon; fs: frameshift.

The second most frequent variation was detected as a C to T SNP at position 194033 only in the field reisolates, resulting in a conservative amino acid substitution at position 210 from alanine to valine in the putative GTPase ObgE protein. Both the alanine and valine amino acids have comparable characteristics and are hydrophobic, nonpolar, and neutral. However, the Ala210Val substitution may result in the alteration of the protein secondary structure at amino acid positions 205 and 215 (Fig. 3A). Another frequent variation in the ObgE region was observed as an A to G SNP at position 193771, and this variation was detected in reisolates collected from vaccinated birds in the field and under controlled conditions. This variation leads to a nonconservative amino acid substitution from arginine to glycine at amino acid position 123. Arginine is a hydrophilic, polar (positively charged), and basic amino acid, whereas glycine is hydrophobic, nonpolar, and neutral. This variation reverts the amino acid sequence of the putative GTPase ObgE protein to that of 7NS and restores the secondary structure of this protein. Another variation is found at the nucleotide position 193771, with a G insertion causing a frameshift at amino acid position 123. This variation was detected in 7 field reisolates, consequently introducing a stop codon at amino acid position 127 and truncating the protein (Fig. 3B). A domain analysis of the ObgE protein by the ScanProsite tool revealed the following regions: an N-terminal domain, residues 1 to 154; a GTP-binding domain (comprised of five conserved motifs, G1 to G5), residues 160 to 331; and a C-terminal domain, residues 350 to 423. The Uniprot analysis revealed that the closest ObgE homologue with its protein function having been experimentally validated is present in Bacillus subtilis with a sequence identity of greater than 40%. The crystal structure of ObgE in B. subtilis was used as a template to predict the 3D structure of ObgE in MS-H and the effect of amino acid substitution on the variants’ stability and solvent accessibility. The Ala210Val substitution has likely resulted in the increased stability of the variant protein, compared to that of MS-H, but no change in solvent accessibility. In contrast, the Arg123Gly substitution (reverting to the 7NS-ObgE genotype) has probably led to a slight reduction in the stability and solvent accessibility of the variant proteins, compared to those of MS-H (Table S4).

**FIG 4:**
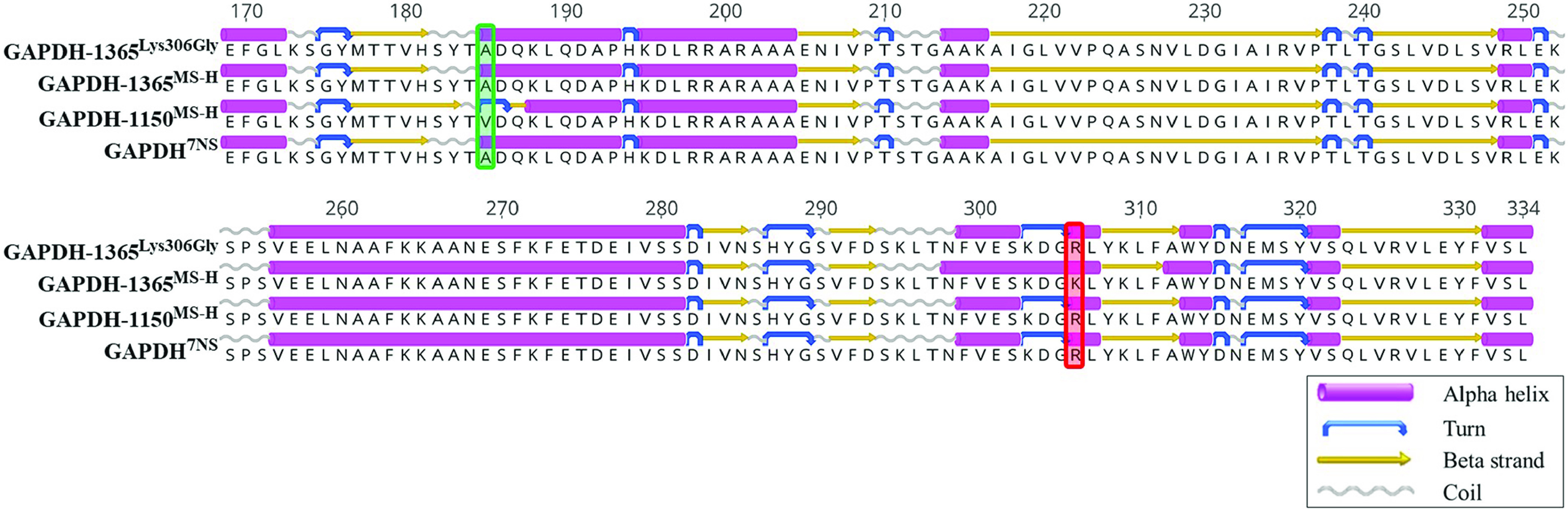
Protein structure analysis of glyceraldehyde-3-phosphate dehydrogenase (GAPDH). The 7NS genome contains two identical copies of GAPDH with alanine at position 185 and arginine at position 306 for both copies. In the MS-H genome, the GAPDH-1150 copy contains valine at position 185 and arginine at position 306, whereas the GAPDH-1365 contains alanine at position 185 and lysine at position 306. A total of 13 MS-H reisolates (GAPDH^Lys306Arg^) contained a Lys306Arg substitution that changed the GAPDH-1365 genotype to that of 7NS. However, these reisolates retained the GAPDH-1150 genotype that was identical to that of MS-H (not shown in this figure). The green box highlights the position 185 of the GAPDH codon containing valine in the GAPDH-1150 of the MS-H and MS-H reisolates but containing alanine in GAPDH-1365 of the MS-H and MS-H reisolates, as well as both copies of GAPDH in 7NS. The red box highlights the position 306 of the GAPDH codon containing lysine in the GAPDH-1365 of MS-H but arginine in GAPDH-1150 of MSH, as well as both copies of the MS-H reisolates and 7NS.

The genome of the M. synoviae wild-type strain 7NS contains two identical copies of the 1,005 bp gene encoding GAPDH positioned on each side of the *vlhA* region. However, these coding regions differ from each other (and from 7NS) in the MS-H genome in that they display a G to A SNP at positions 554 of the MSH_RS01150 (GAPDH-1150) and 917 of the MSH_RS01365 (GAPDH-1365) genes (23). A total of 13 reisolates collected from the field contained an amino acid substitution from lysine to arginine at position 306 of the GAPDH-1365 codon, resulting in an identical amino acid sequence to that of the GAPDH codon in 7NS (Fig. 4). Protein domain prediction detected a 167 aa segment as the nicotinamide adenine dinucleotide (NAD)-binding domain (4 to 170 aa), a 162 aa segment as the C-terminal domain (154 to 315 aa), and an 8 aa active site at position 152 to 159 of the protein. The Site Directed Mutator tool showed that the Lys306Arg substitution is anticipated to have contributed to increased protein stability and solvent accessibility (Table S4). A secondary protein structure analysis showed that the Lys306Arg substitution was likely to alter a 10-amino acid length (aa) alpha helix in the predicted C-terminal domain to a 1 aa coil, a 3 aa turn, and two 4 aa and 2 aa alpha helices (Fig. 4). The Uniprot analysis of the GAPDH of M. synoviae revealed 63% and 62% amino acid sequence identities to the GapA proteins of M. genitalium and M. pneumoniae, respectively.

**FIG 5:**
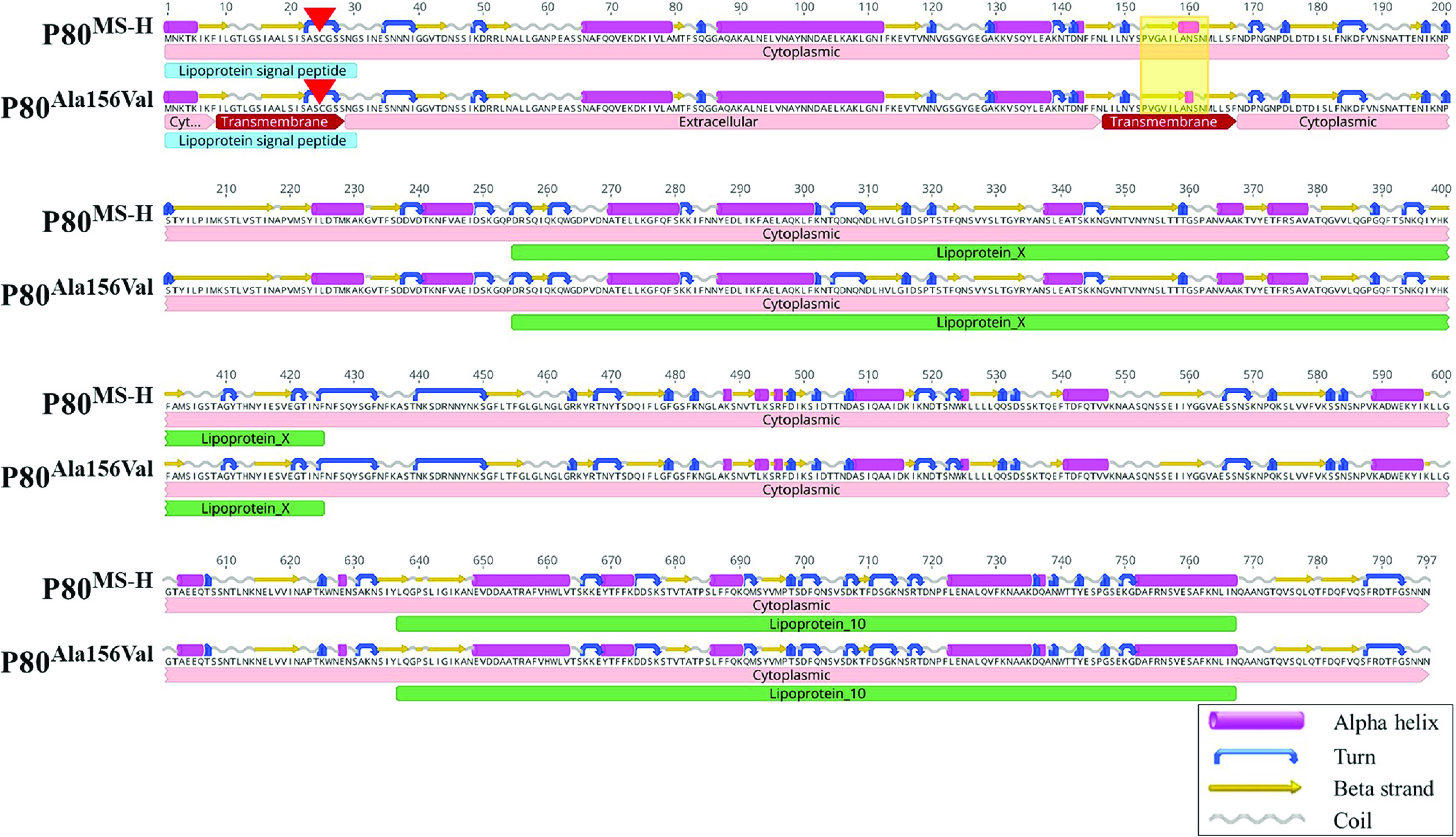
Comparison of predicted secondary structure and topology of the P80 variant containing the Ala156Val substitution that was detected in 11 reisolates to those of the P80 in MS-H. A red arrow indicates the predicted cleavage site between positions 24Ser and 25Cys. The yellow boxes highlight predicted secondary structure changes in the P80^Ala156Val^ variant.

The region MSH_RS01430, coding for a putative P80 family lipoprotein, contained an identical variation in 11 of the reisolates that were collected from commercial vaccine production stages, *in vitro* passages, vaccinated birds under controlled conditions, and in the field. This variation was detected at position 316188 as a SNP and resultedf in an amino acid change from alanine to valine at amino acid 156. Although the Ala156Val substitution was not found to affect the secondary structure of the protein at position 156, it was predicted to affect the secondary structure of the P80 surrounding amino acid 156 (Fig. 5). The signal peptide predictor SignalP identified the amino acids 1 to 30 as a lipoprotein signal peptide of a P80 lipoprotein, with a predicted cleavage site between position 24Ser and 25Cys transported by the secretory (Sec) translocon and cleaved by signal peptidase II. Transmembrane topology prediction was performed using the transmembrane protein topology prediction method based on a hidden Markov model (TMHMM) in Geneious Prime (version 2022.1.1) software, and it was predicted that the P80 protein was likely to be located in the cytoplasm. However, the P80^Ala156Val^ variant was predicted to contain transmembrane regions from amino acids 9 to 28 and 147 to 167, suggesting that the fragment 29 to 146 was likely to be extracellular (Fig. 5). Based on a Uniprot analysis, this protein has 28.1% and 22.7% sequence similarity to the amino acid sequence of a P80 family lipoprotein in M. agalactiae and M. hominis, respectively (25, 26).

## DISCUSSION

In this study, the stability of a live attenuated mycoplasma vaccine was assessed after *in vitro* and *in vivo* passages under different circumstances to complement previous findings regarding the stability of the MS-H genome in a limited number of field reisolates (24).

### Minimal passages *in vitro* do not affect the MS-H genome drastically.

Mycoplasmas retain a stable genome, despite the high frequency of spontaneous mutations in mollicutes (27–30). Mycoplasma synoviae is capable of antigenic variability under laboratory conditions (31). However, the presence of mutations in housekeeping genes after repeated *in vitro* passages has not been investigated systematically. In our study, the genome comparison of MS-H after undergoing 19.92 generations in laboratory conditions with that of the initial MS-H culture led to the detection of three variations. This finding suggests that a limited number of *in vitro* passages with low generation numbers is unlikely to result in and/or reveal spontaneous mutations. A mutation found in a colony obtained from the first stage of vaccine production was also detected in two other colonies selected after minimal *in vitro* passages. While the master seed, working seed, and commercial batches were sequenced directly, the colonies from the first and final stages of vaccine production, also after minimal *in vitro* passages, were obtained after sequential passages onto solid media followed by propagation in liquid media. Therefore, the variations detected in these colonies were likely to be due to the laboratory propagation of the clones. These findings suggest that the MS-H genome is relatively stable within 20 generations. Furthermore, the fact that the genomes of the Vaxsafe MS master and working seeds, as well as those of the commercial batches, are identical indicates that a large-scale production process with the same generation numbers does not result in higher mutation rates, either.

### MS-H undergoes further mutations in birds.

At least one variation was detected in nearly 87% of the MS-H reisolates from the vaccinated field birds examined in this study, 56% of the reisolates from SPF birds under experimental conditions, and 33% of the reisolates after serial passaging in SPF birds. These findings indicate the higher possibility of detection and selection of the spontaneous mutations in the natural host. Interestingly, more than 80% of these variations were detected in noncoding regions, suggesting that the protein structure (as opposed to expression) profile of MS-H is stable after vaccination in chickens. Furthermore, only 5.3% of the predicted coding regions contained variation in more than one genome, signifying that most of these variations occurred rarely (were detected only once) after vaccination. The presence of only one mutation in the vaccinated chickens that were subjected to serial *in vivo* passaging suggests that 2 weeks of incubation in the host was likely inadequate for the domination of the colonized reisolates in the host’s tracheal epithelium. We identified six hotspots in the MS-H genome at which most of the variations between reisolates collected under the various circumstances were preferentially found. These hotspots were located in regions coding for a putative ABC transporter OppF, ObgE, GAPDH proteins, and a P80 lipoprotein.

### Full-length OppF protein is proposed to improve the fitness of MS-H in birds.

The coding region for *oppF* contained variations in 64.3% of the sequenced field reisolates. The amino acid sequence similarity analysis suggests that the full-length protein is a part of an ABC transporter and is expected to be involved in transporting peptides. Due to the reduced genome size and the loss of metabolic pathways through evolution from Gram-positive bacteria, mycoplasmas are dependent on their environment to supply nutrients (32). Most of the variations found in the *oppF* region of the MS-H reisolates resulted in a frameshift which removed a stop codon. The T insertion at position 467 of this coding region is a back mutation to the wild-type genotype in the 7NS genome and has been shown to result in the full-length expression of the OppF protein *in vitro* (24). Therefore, it appears that while the truncated version of OppF in MS-H does not affect the peptide uptake of MS-H under *in vitro* conditions, it is unlikely to be sufficient under the *in vivo* conditions for this purpose. This could be because of the different peptides available in the natural ecological niche compared to the laboratory optimized medium. We propose that the expression of the full-length OppF in the natural host is probably essential for peptide uptake and potentially for MS-H metabolism. Hence, this protein is likely to be involved in MS-H fitness and pathogenesis. It is worth mentioning that many mycoplasma genomes encode several putative nutrient transporters, meaning that their functions are not always reliably predictable based on sequence similarity with proteins encoded by homologous genes in other bacteria and need to be experimentally validated (33).

### Amino acid residues 128 to 423 of ObgE are dispensable.

The region coding a putative ObgE protein (MSH_RS00965) was among the coding regions prone to mutation with detected variants in 55 reisolates collected from vaccinated chickens in the field and under laboratory conditions. The closest ObgE homologue with an experimentally validated function was observed in Bacillus subtilis, where it played roles in the promotion of growth and in stress responses (34, 35). The most frequent variation in the ObgE region in the MS-H genome was detected only in the field reisolates and resulted in an amino acid substitution at position 210. This variation has previously been reported in four field reisolates, three of which had also lost the temperature-sensitive phenotype, suggesting that this mutation might be responsible for the restoration of the wild-type strain 7NS phenotype (36). The amino acid 210 has been proposed to be a part of the conserved putative GTP binding motif G3 of the ObgE protein, and thus, variations in this region may affect the structure and function of this protein (36, 37). The other frequent variation detected in vaccinated chickens under both field and laboratory conditions resulted in an amino acid substitution at position 123 that reverted the genotype to the wild-type strain 7NS. This variation has previously been reported in eight MS-H reisolates with a confirmed loss of the temperature-sensitive phenotype (36). Shahid et al. (2013) suggested that the glycine residue at amino acid position 123 was a highly conserved domain of the ObgE protein and was likely to be significant in the structure and thermosensor functions of this protein (36). However, it has been shown that a single Gly124Arg mutation in ObgE does not produce a *ts^+^* phenotype in *M. mycoides* subsp. *capri* (38). Interestingly, we have detected a higher occurrence of the ObgE^Ala210Val^ variant compared to the ObgE^Arg123Gly^ variant. This observation suggests that in the natural host, a C to T polymorphism is likely to represent a preferential strategy for restoring the *ts*^−^ phenotype compared to an A to G, possibly because of the preferred low guanine-cytosine (GC) percent composition in mollicutes, thereby resulting in a potentially more stable ObgE protein. A total of 7 field reisolates contained a G insertion at the nucleotide position 193771, causing a frameshift at amino acid position 123 and consequently truncating the protein at position 127. Despite the essential role of ObgE in growth in bacteria, a truncated form of this protein can support growth for as long as the N-terminal domain is present (39). In contrast, the deletion of the first five amino acids of ObgE in Escherichia coli and the mutation at position 80 of this protein in Caulobacter crescentus have been shown to inhibit growth (40, 41). The existence of 7 MS-H variants carrying a truncated ObgE protein suggests that the presence of 127 aa in the N-terminal domain is sufficient for growth in M. synoviae and that the remaining part of this protein might be dispensable, confirming previous observations in E. coli (40).

### GAPDH is likely to be a “moonlighting” protein playing a role in pathogenesis.

In 13 of the sequenced reisolates in this study, a hotspot was detected in GAPDH-1365 as an identical variation that resulted in a missense amino acid change in the GAPDH protein. This variation restores the genotype of the GAPDH-1365 copy to that of the wild-type strain 7NS and alters the predicted secondary structure of the C-terminal region. Interestingly, no variation was detected in the GAPDH-1150 copy in any of the 143 sequenced reisolates. The GAPDH protein is commonly found as a tetramer of identical subunits in eukaryotes and prokaryotes and plays an important role in glycolysis as a cytoplasmic protein. The homologous GAPDH protein in M. genitalium, M. pneumoniae, M. hyopneumoniae, Mycoplasma suis, and M. hyorhinis has been shown to contain a surface-localized region and contribute to host cell adhesion (42–46). Although the Lys306Arg substitution found in 13 of the MS-H reisolates is not in the NAD-binding domain of GAPDH, this frequent reversion to the wild-type C-terminal seems to be beneficial for infection. Remarkably, the C-terminal region of GAPDH is the only region confirmed in the localization on the surface of M. pneumoniae with the cytoadhesion function (47), suggesting that M. synoviae GAPDH might be a dual-function protein, with the C-terminal being involved in host-pathogen interaction. Therefore, the GAPDH^Lys306Arg^ variant is proposed to provide a significant advantage in cell adhesion for those MS-H reisolates, as well as for the wild-type 7NS, and thus potentially confers a superior pathogenicity, compared to MS-H. This is consistent with the report by Kordafshari et al. (2020) that this mutation was likely to influence the systemic antibody responses raised by the host against MS-H (24). The absence of any variation in the GAPDH-1150 suggests that this copy could still be useful in providing the enzymatic activity of the GAPDH tetramer, despite its difference from the wild-type version.

### P80 lipoprotein might encounter phase variation in surface display.

Mycoplasma lipoproteins are exposed on the cell surface and are capable of phase variation, which enables mycoplasmas to evade the host immune system (1). A nucleotide variation in the putative P80 family lipoprotein gene (MSH_RS01430) was observed in two colonies obtained after six serial passages *in vitro*, one colony from the first stage of the production of a batch of commercial vaccine, three colonies obtained from vaccinated birds under controlled conditions, and five colonies collected from vaccinated birds in the field. There are three regions in the MS-H genome encoding putative P80 family lipoproteins: MSH_RS01685 with a length of 771 aa and a molecular weight of 83.85 kDa, MSH_RS01655 with a length of 778 aa and a molecular weight of 85.46 kDa, and MSH_RS01430 with a length of 798 aa and a molecular weight of 87.30 kDa. All three of these regions are related to the P80 family and contain a central (lipoprotein X) and a C-terminal (lipoprotein 10) domain, and these domains are present in the family 2 lipoproteins of mycoplasmas. The homologues of the family 2 lipoproteins in M. pneumoniae, M. gallisepticum, and M. hyopneumoniae have been shown to exhibit differential expression during infection and are anticipated to be involved in pathogenesis (48–51). The *in silico* analysis of the protein structure of the P80 variant found in 11 sequenced colonies predicted a possible topology alteration, exposing a 118 aa-length fragment outside the mycoplasma cell. The altered exposure of surface epitopes, known as surface masking, has been observed in the P56 of *M. hominis*, the P29 of M. fermentans, and the Vlp of *M. hyorhinis*, resulting in the phase-variable display of these surface lipoproteins (52–54). The P80^Ala156Val^ variant was also detected in the colonies that were collected after minor *in vitro* passage and from the first stage of vaccine production (see above). Therefore, the P80^Ala156Val^ variants observed in the reisolates collected from vaccinated birds could be produced by the laboratory propagation process, not necessarily by selection pressure in the host.

### Seven coding regions prone to mutations were identified in MS-H.

We have found that the genome of the MS-H strain contains seven coding regions that are prone to mutation (found in 10 or more field reisolates), indicating selection pressure in the host with different ages and nutrition and treatment regimens. The majority of these coding regions are potentially responsible for nutrient uptake and metabolism, such as the OppF ABC transporter being responsible for peptide uptake, GAPDH reversibly converting glyceraldehyde-3-phosphate to 1,3-bisglycerophosphate in glycolysis, and another ABC transporter being responsible for multiple sugar uptake. While OppF and GAPDH are likely to be immunogenic (55–57), there is limited information on the immunogenicity of other proteins that contain variations. Another coding region that is prone to mutation is a putative DNA topoisomerase IV subunit A (ParC) that contains variations that were previously described to correlate with enrofloxacin resistance in M. synoviae (58, 59), suggesting that the farms from which these samples were collected might have not been treated in accordance with the importance rating of this antimicrobial drug. Interestingly, out of 14 reisolates with variations in ParC (correlated with enrofloxacin resistance), 6 were collected from unvaccinated birds in a farm with a history of MS-H vaccination up until 2 years prior to sample collection. In the 2 years preceding sample collection, the farm had implemented enrofloxacin medication instead of MS-H vaccination for the control of M. synoviae infections. Genome analysis of isolates collected from repeated sampling in this farm showed that MS-H persisted in the flocks after the cessation of vaccination. These results suggest that a lateral transmission had occurred from previously vaccinated flocks. The *in vitro* susceptibility profile of one of these six reisolates confirmed resistance against enrofloxacin and difloxacin (data not shown). It is worth mentioning that fluoroquinolones have not been used in commercial poultry farms in Australia, which provides an explanation why none of the Australian isolates carried such mutations. The coding regions ObgE and DNA-directed RNA polymerase subunit beta are proposed to have a role in cell growth, in addition to the correlation of mutations in ObgE and the *ts* phenotype that was described previously (36). Finally, a region coding for a P80 family lipoprotein was detected among the mutation-prone coding regions, and this lipoprotein might be responsible for evasion from the host immune system. 12 genomes contained synonymous codon changes, including 5 passaged reisolates in the TrkH family potassium uptake protein, 1 small-scale progeny in a GNAT family N-acetyltransferase, 3 field reisolates in the sugar ABC transporter, and 3 controlled reisolates in the RNA polymerase sigma factor. While these synonymous codon changes do not affect the protein sequence, using a different codon might have an impact on the translation of the protein, possibly because of the different concentrations of the isoacceptor tRNAs in low GC organisms, such as mycoplasmas (60, 61).

In conclusion, this study used MS-H as a model to deliver a comprehensive investigation of the mutation frequencies of mycoplasmas under various *in vitro* and *in vivo* conditions. Future *in vivo* studies are needed to assess the effects of these variations on the virulence and immunogenicity of MS-H and, potentially, other mycoplasma pathogens.

## MATERIALS AND METHODS

### Culture medium.

The MS-H vaccine and its reisolates were grown in mycoplasma broth (MB) or mycoplasma agar (MA) containing 10% swine serum (Sigma-Australia) and 0.01% NAD (Sigma-Australia), based on the formulation of Frey’s medium with minor modifications (62, 63). The MB cultures were incubated at either 33°C or 37°C until the late logarithmic phase (approximate pH value of 6.8) (64). To eliminate the cultivation process bias in mutation selection, filter-cloning was avoided, and sequential passaging from the clone obtained from the MA was limited.

### Field reisolates of MS-H.

The MS-H field reisolates used in this study were obtained from tracheal swabs collected from poultry flocks in 10 countries from 1993 to 2020 (Table S1). All of the birds had been exposed to MS-H or had been vaccinated at 3 or 4 weeks of age, and the specimens were collected between 7 and 71 weeks after vaccination. The tracheal swabs were inoculated into appropriate media and were cultivated using standard methods. The distinct colonies were subjected to genotyping using *vlhA*, *oppF*, and/or *obgE* sequencing (65–67) via multilocus variable number of tandem-repeats analysis (MLVA) (68) or multilocus sequence typing (MLST) (69). The confirmed MS-H reisolates were sent to our laboratory as pure cultures and were stored at −80°C for further processing. For DNA extraction and whole-genome sequencing, the cultures were thawed, and 200 μL were inoculated in 40 mL MB and were incubated at 37°C until the late logarithmic phase (Table S2).

### Samples of MS-H from the Vaxsafe MS vaccine.

The Vaxsafe MS master and working seeds, which are used to produce the commercial MS-H vaccine, were provided by Bioproperties Pty., Ltd. Moreover, four randomly selected commercial vaccine vials produced in 2007 (batch number 072991A), 2010 (batch number 102681A), 2016 (batch number 161071A), and 2020 (batch number 202071BG) were included in the study. A total of 1 mL from each master seed, working seed, or commercial vaccine vial was used for nucleic acid extraction. Finally, industrial fermenter samples from the first and last stages of production of a commercial vaccine batch made in 2020 were transferred onto MA and were grown at 37°C for 10 days. Three distinct colonies, designated large-scale progenies, from each stage were selected and grown in 40 mL MB and incubated at 37°C until the late logarithmic phase (Table S2).

### *In vitro* passage of MS-H.

To evaluate the effect of *in vitro* passage on MS-H mutations, an aliquot of the MS-H strain that was previously sequenced in our laboratory (UoM_MS-H, GenBank accession number CP021129) was passaged *in vitro* six times at a 1:10 dilution in a final volume of 10 mL of MB and incubated at 33°C (optimum growth temperature) until the late logarithmic phase. Since each 1:10 dilution would result in 3.32 generations of MS-H, the 6 total passages are predicted to produce 19.92 generations of MS-H. The final passage was transferred onto MA and was incubated at 33°C for 10 days. Five distinct colonies, designated small-scale progenies, were selected and grown in 40 mL of MB and were incubated at 33°C until the late logarithmic phase (Table S2).

### *In vivo* passage of MS-H strain.

MS-H was passaged *in vivo* through 5 passages in chickens as described previously (14) (Fig. S1). Briefly, 5 two-week-old, specific-pathogen-free chickens were vaccinated via eyedrop with 50 μL of MS-H (batch number 250294-2) and were kept in isolators for 2 weeks. The birds were then euthanised at 4 weeks of age, and their tracheas were removed. The tracheal washing was prepared by aspirating 5 mL of MB through the trachea 10 times. Nasal turbinates were added to the tracheal washings and vortexed vigorously. Serial 10-fold dilutions of the mixtures were incubated at 33°C until the late logarithmic phase. A total volume of 50 μL from the lowest dilution was used for eyedrop administration into the next five chickens. The remaining culture from the lowest dilution of the tracheal washing/nasal turbinate mixtures from five chickens were transferred onto MA and were incubated at 37°C for 10 days. This process was repeated through five chicken passages. A total number of 15 colonies (3 collected from each passage) were selected from the MA plates and were grown in 40 mL of MB and incubated at 37°C until the late logarithmic phase. The MS-H vaccine strain used in this experiment was also grown in 40 mL of MB and incubated at 37°C until the late logarithmic phase (Table S2).

### Vaccination of SPF chickens under controlled conditions.

5 four-week-old chickens were vaccinated via eyedrop with a 0.1 mL dose of the MS-H working seed (BPL-157), which contained 10^7.6^ color changing units (CCU) (equivalent to the registered maximum release titer of 10^9.1^ CCU/mL in Australia) (Fig. S2). Swabs from the upper, middle, and lower trachea were taken from 5 chickens after 60 days postvaccination, immediately inoculated onto MA plates, and incubated at 37°C for 7 days. A total of 25 colonies (5 per bird) were grown in 40 mL of MB and incubated at 37°C until the late logarithmic phase. The MS-H vaccine strain used in this experiment was also grown in 40 mL of MB and incubated at 37°C until the late logarithmic phase (Table S2).

### DNA extraction, library preparation and sequencing.

Cells were collected from 40 mL cultures or 1 mL vaccine vials via centrifugation at 10,000 × *g* for 30 min at 4°C, and this was followed by two steps of washing with 1 mL phosphate-buffered saline (PBS). The DNA was extracted using Qiagen's DNeasy Blood and Tissue Kit, according to the standard protocol for Gram-negative bacterial cells. DNA was eluted in 10 mM TrisHCl (pH 8.0). The integrity of the DNA was confirmed on a 1.0% agarose gel that contained SYBR safe gel stain. The concentration of the samples was determined using a Qubit high sensitivity assay, and Nanodrop was used to assess sample purity. Sample concentrations were normalized to 10 ng per μL with 10 mM Tris (pH 8.0). In the next step, 100 ng of extracted DNA was used to prepare sequencing libraries using Illumina's Nextera Flex DNA Library Prep Kit. Samples were indexed with 8 bp, unique dual indices, using 6 polymerase chain reaction (PCR) cycles. Sequencing was performed on the Illumina MiSeq or NovaSeq platforms, using paired-end 300 bp or 150 bp reads, respectively, at the Deakin Genomics Centre, Victoria, Australia.

### Genomic analysis.

The Illumina reads were processed using Trimgalore to trim the bases below a Phred quality value of 25 and remove the Nextera adapter sequences. This was followed by a confirmation of the quality of the filtered reads using FastQC. The reads were aligned to the MS-H sequence (GenBank accession number CP021129) for single nucleotide polymorphism and insertion/deletion (indel) analyses using Snippy (70). The key parameters of variant calling by Snippy included a minimum number of 10 reads of coverage to consider variant calling, and at least 5% of those reads had to differ from the reference in order to call a variation. The highly repetitive, variable, and similar regions of the genomes, such as the *vlhA* gene and pseudogenes, the IS1634 family transposase, and the type III restriction endonuclease subunit M regions, were excluded from the variation assessment.

### Protein structure and function analyses.

The protein structure homology-modeling tool SWISS-MODEL was used to build the models and predict the protein 3-dimensional (3D) structures (71). The effects of variations on the stability and the secondary structures of proteins were assessed using Site Directed Mutator (72) and Geneious Prime 2021.1.1 (www.geneious.com), respectively. The UniProt web server was used to search for homologous proteins in order to predict the functions of proteins (www.uniprot.org). The ScanProsite tool (https://prosite.expasy.org/scanprosite) and the SUPERFAMILY database (www.supfam.org) were used to predict the protein domains. The Phobius database was used to predict the protein topology (https://phobius.sbc.su.se). The SignalP 5.0 was used to predict the presence of signal peptides and the locations of their cleavage sites in proteins (https://services.healthtech.dtu.dk/service.php?SignalP-5.0).
