# Supplementary material for: Genomic Diversity of a Globally Used, Live Attenuated Mycoplasma Vaccine
Source: Microbiol Spectr. 2022 Nov 1;10(6):e02845-22. doi: 10.1128/spectrum.02845-22 (PMC9769879; doi:10.1128/spectrum.02845-22)
Supplement: Supplemental file 1 — Eig. S1 and S2 and Tables S3 to S4. Download spectrum.02845-22-s0001.pdf, PDF file, 0.4 MB [file spectrum.02845-22-s0001.pdf]

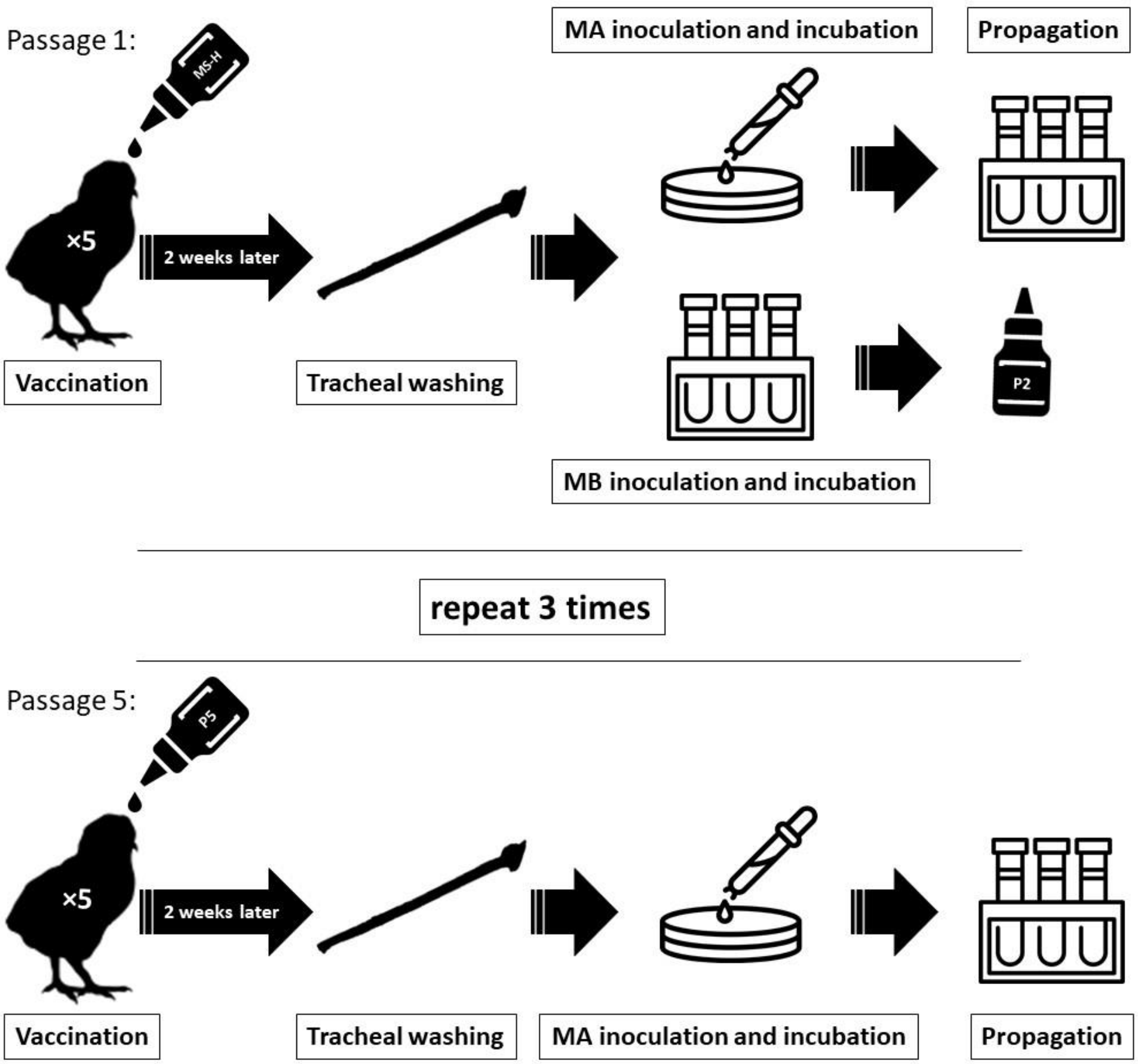

Figure S1. Schematic figure of the *in vivo* passage of MS-H strain five times: Briefly, five 2-weeks-old specific-pathogen-free chickens were vaccinated by eyedrop with 50µL of MS-H. The birds were then euthanised at 4 weeks of age and the tracheas were removed. Tracheal washing was prepared by aspirating 5 ml of MB through the trachea for 10 times. The nasal turbinates were added to the tracheal washings and vortexed vigorously. Serial ten-fold dilutions of the mixtures were incubated at 33°C until late logarithmic phase. A total volume of 50µL from the lowest dilution was used for eyedrop administration to the next five chickens. The remaining culture from the lowest dilution of washing/nasal turbinates mixtures were transferred onto MA and incubated at 37°C for 10 days. This process was repeated through five chicken passages. A total number of 15 colonies (three per passage) were selected from MA plates and grown in 40 mL MB and incubated at 37°C until late logarithmic phase.

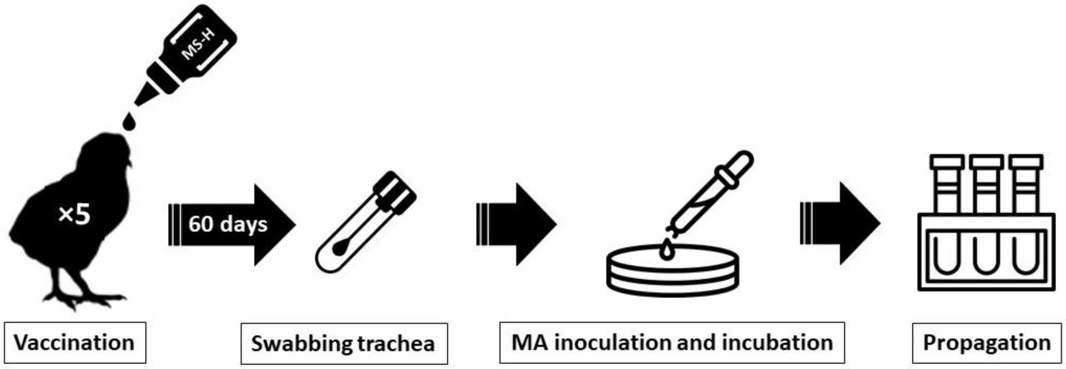

Figure S2. Schematic figure of the laboratory simulation of on-farm vaccination: A total of five four-week-old chickens were vaccinated with 0.1 mL dose of the MS-H vaccine, containing $10^{7.6}$ colour changing units (CCU) by eye drop (equivalent to the maximum release titre of $10^{9.1}$ CCU/mL). Swabs from upper, middle and lower trachea were taken from five chickens after 60 days post vaccination and inoculated immediately onto MA plates and incubated at 37°C for 7 days. A total number of 25 colonies (five per bird) were grown in 40 mL MB and incubated at 37°C until late logarithmic phase. The MS-H vaccine strain used in this experiment was also grown in 40 mL MB and incubated at 37°C until late logarithmic phase.

Table S1. History of MS-H field reisolates (if available)

Accession	Host species	Host type	Sample collection year	Age of host	Country of origin	Comment
AB1	chicken		1993	28 weeks	Australia	
AS2	chicken		1993	11 weeks	Australia	
TS3	chicken		1993	14 weeks	Australia	
TS4	chicken		1994	28 weeks	Australia	
TS5	chicken		1994	37 weeks	Australia	
TS6	chicken		1994	55 weeks	Australia	
I45	chicken		2014	59 weeks	Italy	Isolate IZSVE/2014/368-2*
I46	chicken		2014	59 weeks	Italy	Isolate IZSVE/2014/368-6*
I47	chicken		2014	59 weeks	Italy	Isolate IZSVE/2014/374-11*
I48	chicken		2014	59 weeks	Italy	Isolate IZSVE/2014/374-12*
M152	chicken	Broiler	2015		Hungary	
M153	chicken	Broiler	2015		Hungary	
M155	chicken	Broiler	2015		Hungary	
M157	chicken	Broiler	2015		Hungary	
M163	chicken	Broiler	2015		Hungary	
M200	chicken	Broiler	2015	50 weeks	Hungary	
M214	chicken	Layer	2015	63 weeks	Hungary	
M215	chicken	Layer	2015	63 weeks	Hungary	
M235	chicken	Breeder	2015	47 weeks	Hungary	
M236	chicken		2015	35 weeks	Hungary	
M239	chicken	Breeder	2015	63 weeks	Hungary	
M240	chicken	Breeder	2015	35 weeks	Hungary	
I01	chicken	Breeder	2015	60 weeks	Italy	
I02	chicken	Breeder	2015	39 weeks	Italy	
I03	chicken	Breeder	2015	42 weeks	Italy	
I04	chicken	Breeder	2015	18 weeks	Italy	
I05	chicken	Breeder	2015	18 weeks	Italy	
I06	chicken	Breeder	2015	44 weeks	Italy	
M254	chicken	Layer	2016	71 weeks	Hungary	
M272	chicken		2016		Ukraine	
M289	chicken		2016		Hungary	
M306	turkey		2016		Hungary	off-label use under veterinary direction
M308	turkey		2016		Hungary	off-label use under veterinary direction
I07	chicken	Breeder	2016	31 weeks	Italy	
I08	chicken	Breeder	2016	31 weeks	Italy	

I09	chicken	Breeder	2016	31 weeks	Italy
I10	chicken	Breeder	2016	31 weeks	Italy
I11	chicken	Breeder	2016	31 weeks	Italy
I12	chicken	Breeder	2016	31 weeks	Italy
I13	chicken	Breeder	2016	31 weeks	Italy
I14	chicken	Breeder	2016		Italy
I15	chicken	Layer	2016	30 weeks	Italy
I16	chicken	Breeder	2016		Italy
I17	chicken	Breeder	2016		Italy
I18	chicken	Breeder	2016		Italy
I23	chicken	Layer	2016		Italy
M376	chicken		2017	16 weeks	Romania
M378	chicken		2017	17 weeks	Romania
M379	chicken		2017	15 weeks	Romania
M380	chicken		2017	13 weeks	Romania
M381	chicken		2017	22 weeks	Romania
I19	chicken	Breeder	2017	27 weeks	Italy
I20	chicken	Breeder	2017		Italy
I21	chicken	Layer	2017		Italy
I22	chicken	Broiler	2017		Italy
I24	chicken	Layer	2017	19 weeks	Italy
I25	chicken		2017		Italy
I26	chicken	Layer	2017	40 weeks	Italy
I27	chicken	Breeder	2017	40 weeks	Italy
I28	chicken	Breeder	2017	40 weeks	Italy
M510	chicken		2018		Slovakia
M516	chicken	Broiler Breeder	2018	26 weeks	India
M517	chicken		2018	23 weeks	India
M528	chicken		2018		China
M529	chicken		2018		China
M542	chicken		2018	16 weeks	India
M544	chicken	Broiler Breeder	2018	22 weeks	India
M545	chicken	Broiler Breeder	2018	22 weeks	India
I29	chicken	Breeder	2018	8 weeks	Italy
I30	chicken	Breeder	2018	6 weeks	Italy
I31	chicken	Layer	2018	7 weeks	Italy
I32	chicken	Layer	2018	9 weeks	Italy
I33	chicken	Layer	2018		Italy
I34	chicken	Layer	2018	12 weeks	Italy
I35	chicken	Layer	2018		Italy
I36	chicken	Layer	2018	22 weeks	Italy
I37	chicken	Layer	2018	22 weeks	Italy
I38	chicken	Breeder	2018		Italy
I39	chicken	Breeder	2018		Italy

M594	chicken		2019	15 weeks	Romania	
M618	chicken	Layer	2019	50 weeks	Romania	
A-A	chicken		2019		Argentina	Not vaccinated
A-B	chicken		2019		Argentina	Not vaccinated
A-C	chicken		2019		Argentina	Not vaccinated
A-D	chicken		2019		Argentina	Not vaccinated
A-E	chicken		2019		Argentina	Not vaccinated
A-F	chicken		2019		Argentina	Not vaccinated
I41	chicken	Breeder	2019		Italy	
I42	chicken	Breeder	2019		Italy	
I43	chicken	Breeder	2019	32weeks	Italy	
AQJ5A	chicken		2019		Australia	
AQJ5B	chicken		2019		Australia	
AQJ9B	chicken		2019		Australia	
N01	turkey		2019		Netherlands	off-label use under veterinary direction
N02	turkey		2019		Netherlands	off-label use under veterinary direction
N03	chicken	Broiler Breeder	2020		Netherlands	
N04	chicken	Broiler Breeder	2020		Netherlands	
N05	chicken	Broiler Breeder	2020		Netherlands	

*Isolates have been used in the following study: Moronato et al., 2018. Application of different laboratory techniques to monitor the behaviour of a *Mycoplasma synoviae* vaccine (MS-H) in broiler breeders. Vet. Res. 14:1-9.

Table S2. Details of clone preparation in this study

Experiment type	Clone name	Number of passages	Culture volume	Number of selected colonies	Colony propagation volume
In vitro	Small-scale progeny	6	10 mL	5	40 mL
	Large-scale progeny	6	commercial fermenter	6	40 mL
In vivo	Field reisolate	1	NA	98	Pure culture was provided
	Controlled reisolate	1	NA	25	40 mL
	Passaged reisolates	5	NA	15	40 mL

Table S3. Coding regions containing variation(s) in more than one field reisolate

Locus-tag	Product	Number of genomes containing variation(s) (/98)
**MSH_RS01740**	**ABC transporter, OppF**	62
**MSH_RS00965**	**GTPase, ObgE**	54
**MSH_RS01365**	**Glyceraldehyde-3-phosphate dehydrogenase, GAPDH**	13
**MSH_RS00320**	**DNA topoisomerase IV subunit A, ParC**	14
MSH_RS01430	P80 family lipoprotein	12
**MSH_RS02465**	**DNA-directed RNA polymerase subunit beta**	12
**MSH_RS00575**	**Sugar ABC transporter**	10
MSH_RS03140	M42 family metallopeptidase	8
MSH_RS00255	Hypothetical protein	7
MSH_RS01685	P80 family lipoprotein	7
MSH_RS03070	Type IIA DNA topoisomerase subunit B	7
MSH_RS02015	Thymidylate synthase	6
MSH_RS02845	Hypothetical protein	5
MSH_RS03170	Lysine--tRNA ligase	3
MSH_RS02670	Phosphoenolpyruvate--protein phosphotransferase	3
MSH_RS03065	Serine--tRNA ligase	3
MSH_RS02470	DNA-directed RNA polymerase subunit beta	3
MSH_RS02775	Hypothetical protein	3
MSH_RS01875	SGNH/GDSL hydrolase family protein	2
MSH_RS01920	Transcription elongation factor GreA	2
MSH_RS03630	Translation initiation factor IF-2	2
MSH_RS02945	tRNA pseudouridine synthase B	2
MSH_RS02350	tRNA-Asp	2
MSH_RS02560	ECF transporter S component	2
**MSH_RS01000**	**P80 family protein**	2
MSH_RS01195	Hypothetical protein	2
MSH_RS01640	Hypothetical protein	2
MSH_RS00140	Glycerophosphodiester phosphodiesterase	2
MSH_RS02780	UvrD-helicase domain-containing protein	2
MSH_RS02980	Cation-translocating P-type ATPase	2
MSH_RS03180	ABC transporter permease	2
MSH_RS00390	Putative immunoglobulin-blocking virulence protein	2

MSH_RS01090	PDxFFG protein	2
MSH_RS01715	Cell division protein FtsZ	2
MSH_RS02600	ABC transporter ATP-binding protein	2
MSH_RS03505	DAK2 domain-containing protein	2
MSH_RS03580	Restriction endonuclease subunit S	2
MSH_RS03610	PTS ascorbate transporter subunit IIC	2
MSH_RS03360	Hypothetical protein	2

Table S4. Effect of amino acid substitution on stability and solvent accessibility of the proteins coded by mutation-prone regions

Product	Template code used for modelling	Crystal structure source	Global model quality estimation (GMQE)	Sequence identity	Variation(s)	Solvent accessibility[a] (%)	Predicted pseudo ΔΔG[b] (kcal/mol)
GAPDH	7jwk.1.A	*Mycoplasma genitalium*	0.84	63.72%	Lys306Arg	+30.2 to +38	+0.13
ObgE	1lnz.2.A	*Bacillus subtilis*	0.52	48.18%	Arg123Gly	21.2 to 17.2	-0.02
					Ala210Val	0.4 to 0.4	0.57
ParC	2nov.2.A	*Streptococcus pneumoniae*	0.39	47%	Asp84Asn	48.4 to 54.2	-0.53
					Thr85Ile	101.4 to 99.6	1.07
					Asp89Asn	59.9 to 5.3	-0.94

[a] Solvent accessibility of amino acid side chain was from reference (MS-H) to the variant; values less than 17% show inaccessible/buried side chain, values between 17 to 43% show partially accessible side change and values more than 43% is accessible.

[b] Delta G (ΔΔG) is the change in Gibbs free energy between the folded and unfolded states (ΔGfolding) when a point mutation is present; A negative ΔΔG value corresponds to mutation predicted to be destabilising the protein whereas a positive ΔΔG value corresponds to mutation predicted to be stabilising the protein.

Note: only proteins with an available and acceptable quality and identity template were assessed.
